# Microfluidic Paper-Based Analytical Devices (μPADs) and Micro Total Analysis Systems (μTAS): Development, Applications and Future Trends

**DOI:** 10.1007/s10337-013-2413-y

**Published:** 2013-02-22

**Authors:** Piotr Lisowski, Paweł K. Zarzycki

**Affiliations:** Section of Toxicology and Bioanalytics, Koszalin University of Technology, Śniadeckich 2, 75-453 Koszalin, Poland

**Keywords:** Microfluidic paper-based analytical devices (μPADs), Micro total analysis systems (μTAS), Micro-chip chromatography, Micro-planar chromatography (micro-TLC), Detection systems, Biochemical analysis

## Abstract

Microfluidic paper-based analytical devices and micro total analysis systems are relatively new group of analytical tools, capable of analyzing complex biochemical samples containing macromolecules, proteins, nucleic acids, toxins, cells or pathogens. Within one analytical run, fluidic manipulations like transportation, sorting, mixing or separation are available. Recently, microfluidic devices are a subject of extensive research, mostly for fast and non-expensive biochemical analysis but also for screening of medical samples and forensic diagnostics. They are used for neurotransmitter detection, cancer diagnosis and treatment, cell and tissue culture growth and amplification, drug discovery and determination, detection and identification of microorganisms. This review summarizes development history, basic fabrication methods, applications and also future development trends for production of such devices.

## Introduction

Over the past 20 years, there is a rapid development and increasing interest of microfluidic devices also called a micro total analysis system (μTAS), lab-on-chip (LOC) or microfluidic paper-based analytical devices (μPADs) [1, 2]. The ability to perform laboratory operations on nano- or pico-scale, using miniaturized equipment (laboratory glass, laboratory reactors) has opened new ways in modern analytical chemistry, medicine, genetic, cell biology and many other research areas. Manipulation of small volumes of fluids using channels with dimension of tens to hundred of micrometers is very appealing and has been regarded as the most powerful advantage of lab-on-chip [3]. Recently, chemists apply mini-laboratories to synthesize new molecules or materials. Biologists use them to study complex cellular processes in the extensive study of many areas of cell biology. For analytical chemists, microfluidic devices are convenient tools for detection and determination of many organic and inorganic compounds. These simple devices offer analytical and diagnostic abilities that could revolutionize medicine and pharmaceutical industry. They are small, light, portable, and have low manufacturing, usage and disposal costs. Specific to the field of microfluidics is the benefit of low consumption of reagents and analytes [4, 5]. They have been used for wide range of practical applications in many research fields: biomedical science, genomics, forensics, toxicology, immunology, environmental studies, chemistry or biochemistry. Up to this date, microfluidics were successfully used in clinical analysis of blood [6–9], to detect and identify pathogens, proteins [10–13] and environmental contaminants [14–16], in genetic research [17, 18] and drug industry [19–21]. In developing countries, miniaturized portable medical diagnostic tools are especially important for the people having no direct access to medical laboratories with basic diagnostic and analytical facilities [3].

## History and Development of Microfluidic Devices

It is assumed that the first microfluidic device was developed in 1975 [22, 23]; however, some of them evolved from separation techniques based on thin-layer and gas chromatography. In 1938, Ukrainian scientists N.A. lzmailov and his student M.S. Shraiber published the article “Spot chromatographic method of analysis and its applications in pharmacy” in the journal Farmatsiya (Pharmacy) [24]. They were searching for appropriate methods for the rapid analysis of plant extracts. They coated microscope slides with a suspension of various adsorbents (calcium, magnesium, and aluminum oxide), deposited one drop of the sample solution on this layer and added one drop of the solvent. The separated sample components appeared as concentric rings that fluoresced in various colors under a UV lamp, and from that reason one of their conclusions was described as follows: “*A spot chromatographic method of analysis was developed; this method consists in that the separation of substances into zones is observed in thin layers of adsorbents using a drop of the substance*” [25]. In 1947 T.I. Williams described a further improvement of the method of Izmailov and Shraiber [26]. He prepared the adsorbent-coated glass plates in the form of a sandwich, where the adsorbent layer was covered by a second glass plate with a small hole through which the sample (and solvent) drops could be applied. For years, simplified methodology called micro planar chromatography was frequently applied for efficient separation and quantification of inorganic and organic substances [27–31]. Most recently, thermostated micro-TLC protocols were successfully applied for qualitative and quantitative analysis, fractionation, screening and fingerprinting of highly organic compounds loaded biological and environmental samples [32–38]. First publication concerning construction of microfluidic separation device based on miniaturized gas chromatograph and involving integrated circuit processing technology was published in 1975. It consisted of a 5-cm-diameter silicon wafer with an open-tubular capillary column, two sample injection valves and a thermal conductivity detector. This separation device was able to separate a simple mixture of compounds in a matter of seconds [22, 23]. No further research on given miniaturized gas chromatographs was initiated until the 1990s. The response of the scientific community to this first silicon chip device was virtually none, presumably because of the lack of technological experience and the research work focusing on fabrication of the key components like micropumps, microvalves and chemical sensors [1]. Progress in molecular biology greatly stimulated development of capillary electrophoresis for the separation and analysis of DNA and proteins. Also, similar to advances with integrated circuits in electronic and computer industry, biological and chemical analysis devices miniaturization efforts have been made. In 1990, Manz and co-workers presented a miniaturized open-tubular liquid chromatograph using silicon chip technology [39]. Manz is also author of the concept of “miniaturized total chemical analysis system” or μTAS [5]. In his article, he presented a silicon chip analyzers incorporating sample pretreatment, separation, and detection devices and disclosed the ideas of integrating a capillary electrophoresis setting onto a chip. In the last two decades, development of new microfabrication techniques and materials, separation and detection methods (see Table 1) used in μTAS devices was very fast and it is widely described [1, 40, 41]. Currently, the concept of paper-based analytical devices (μPADs) was originated. The first one was invented and described by Whitesides Group of Harvard University in 2007 [42] but its origins date back to the 1940s of the last century, to the work of Müller and Clegg [43], excluding paper strips for the determination of pH. They used a filter paper, made a wax barrier on it and observed that the restricted channel sped up the pigments sample diffusion process, amount of sample consumption and micrograms separation order of magnitude [43]. In recent times, μPADs are widely used in health diagnostics, biochemical analysis, forensic and food quality control [44].

**Table 1:** Basic materials and components for assembling of typical micro total analysis systems (μTAS) working under different separation and detection modes

μTAS fabricaton method	Device body materials	Separation processes	Detection systems	Device main components
Molding [147, 152]	Polymers (PDMS [21, 48, 147, 160, 161] PMMA, [149, 151, 153, 156, 157], PC [152, 156, 157] COC [157, 159, 172] SU-8 [154, 155, 162]	Capillary electrophoresis [152, 160–162, 175]	Conductometry [39]	Inlets, outlets, connectors, microchannels, microchambers [21, 160]
Micromolding in capillaries (MIMIC) [147]	Ceramic [147]	Micellar electrokinetic chromatography [165, 168]	Laser-induced fluorescence [160, 164]	Valves, pumps [21, 57, 160]
LIGA **(Li**thographie, **G**alvanoformung, **A**bformung) [149, 150, 155]	Glass [39, 119, 147, 161, 169]	Capillary electrochromatography [172–175]	Electrochemical [161, 162]	Mixers [64, 160, 176]
Etching [148, 169]	Silicon [23, 39, 119]	Gas chromatography [23]	Fluorescence [21]	Electromagnets [65]
Lithography [21, 48, 160, 162]	Quartz [148]	Liquid chromatography [39, 175]	Absorbance [120, 163]	Microheaters [66]
Phase-changing sacrificial layers (PCSL) [151]		Solid-phase extraction [166]	Atomic fluorescence spectrometry (AFS) [165]	Droplet and bubble generators [67, 69, 96]
Imprinting [153, 160]		Isotachoforesis [169]		
Injection molding [156, 157]			Chemiluminescence [167]	
Conventional machining [158]		Isoelectric focusing [170, 171]	Normal Raman spectroscopy [169]	
Laser ablation [181, 182]			Mass spectrometry [177–180]	
Hot embossing [182, 183]				

## Components of Microfluidic Devices (μTAS)

Generally, considering the publications dealing with the development of microdevices, we can see them clearly divided into two groups. The first one (μTAS) contains the microfluidic devices manufactured by variety of methods and materials that are connected to the external units such as to a sampling unit, detector unit and electronic unit [3]. The second type of microfluidic devices include a cheap, simple in production, paper-based laboratory chips, which in themselves are fully equipped laboratory unit, designed to perform specific tasks, mainly for the detection of various types of substances [44].

The main components of the microfluidic units from the first group are injectors, channels, pumps, valves, storage containers, mixers, electromagnets, microheaters, droplet and bubble generators [1, 3] (see Table 1). Manufacturing methods used for LOC devices were developed in the semiconductor industry [45]; therefore, substrates such as silicon, glass (first microfluidic devices were made in silicon and glass [39]), quartz, soft or hard polymers are used in their production (see Table 1). In addition, various biomaterials including calcium alginate, cross-linked gelatin or hydrogels were applied. They have been useful to provide a physiologically relevant cellular microenvironments that are necessary to cell and tissue culture growth [46, 47]. Silicon is chemically and thermally stable, and channels can be fabricated in this material by etching or photolithography [48]. However, silicon wafers are expensive, brittle and opaque in the UV and visible regions and are therefore not suitable for devices where detection is based on optical sensors [49]. Glass was an early replacement for silicon, being less expensive, transparent, negatively charged, and a good support for electroosmotic flow [50]. Polymers are inexpensive, which allow for the manufacture of disposable devices. They also offer interesting properties for microfluidic devices. Elastomeric materials have good structural rigidity and strength and enable the fabrication of small and rigid microfluidic devices. Channels can be formed in polymers by moulding and various soft lithography processes and sealing of discrete parts can be achieved thermally or with adhesives. However, their surface chemistry is more complex than that of silicon or glass. They are often incompatible with organic solvents and cannot be used at high temperatures [48]. Etching in quartz and glass is also too expensive and time-consuming [48] in comparison to other materials like polymers, photopatternable silicone elastomers, thermoset polysters, poly(methylmethacrylate) (PMMA), poly(dimethylsiloxane) (PDMS), polyamide (PA), cyclic olefin copolymer (COC) and SU-8 (negative photoresist). Applications of above-mentioned microfluidics body materials, which are currently most commonly used materials, enable to minimize the cost of microfabrication [51].

## Flow Control in μTAS

**Fig. 1:**
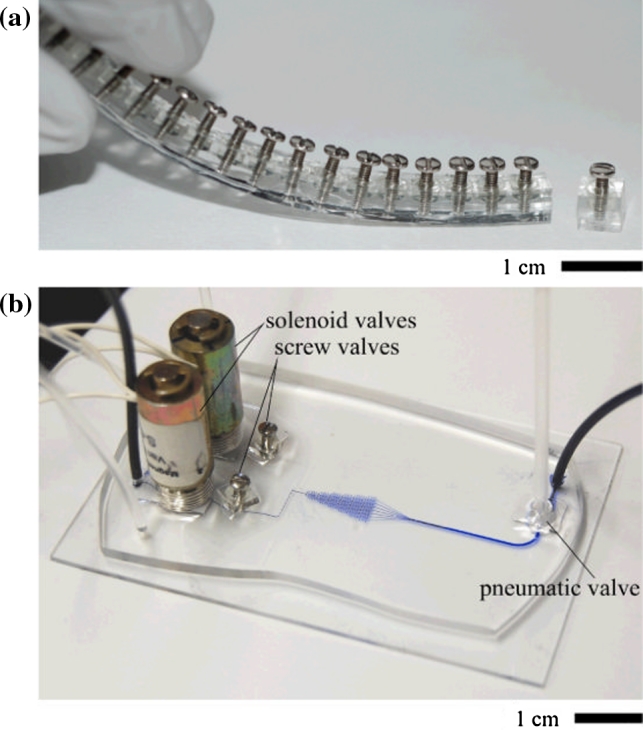
**a** General view of a strip of prefabricated screw valves. A single valve has been separated from the strip using a razor blade. **b** Microfluidic gradient generator containing two embedded solenoid valves, two embedded screw valves and one embedded pneumatic valve (by Hulme et al. 2009 [58]; reproduced by permission of the Royal Society of Chemistry)

Fluid transport and flow in microfluidic devices is achieved and controlled by passive (surface tension, capillary forces) or active (generally pumping—with electrokinetic, electrohydrodynamic, magnetohydrodynamic, electroosmotic pumps; centrifugal force,) mechanisms [52, 53] and is defined by Reynolds number (Re), which is the ratio of the active forces (inertial forces) to the passive forces of internal friction in the fluid, appearing as a dynamic viscosity:

$$ \text{Re} = \frac{\rho vd}{\mu } $$

where *ρ* is the density of the fluid (kg/m^3^), *v* is the mean fluid velocity (m/s), *d* is the channel diameter and *μ* the dynamic viscosity of the channel (kg/m*s). When Re < < 1, the flow is laminar and very smooth. When Re > 10^3^, the flow is turbulent and mainly characterized by vortices [54]. Flow within microstructures typically has Reynolds numbers of 10^−3^–10^−5^ and is characterized by a laminar flow. Contrary to fluid dynamic in larger scales devices, in microfluidic devices viscous forces dominate and turbulences are non-existent. Surface tension can be an important driving force and mixing is slow and occurs through diffusion [55]. Microvalves allow controlling flow and can be segregated in two categories: passive valves (which do not require mechanical actuation) and active valves (which do) (Fig. 1). Typical passive valves are cantilever valves, diaphragm valves, diffuser/nozzle valves [56]. Actuation of active valves is generally piezoelectric, thermopneumatic, electrostatic and electromagnetic, but pneumatically actuated, flexible membrane-based valves (and pumps) are most popular forms of active elements in microfluidic devices [56, 57].

**Fig. 2:**
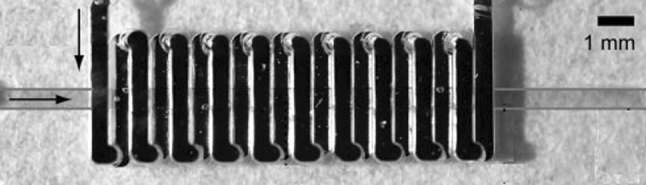
Microheater incorporated in polymer-based (PDMS) microfluidic systems (by Siegel et al. 2007 [66])

For many biological and chemical applications, mixing of transported fluids in microchannels is very important. Mixers are therefore essential in enhancing mixing efficiency and for rapid homogenization of the reagents. All mixing ultimately occurs due to molecular diffusion and therefore the basic idea is reducing the distance over which mixing must occur [59]. They can be classified as active (needed external energy) or passive (mixing in specific geometry of the channel). Passive mixers are usually easier to fabricate than active mixers and are more suitable for applications [60]. Typical active mixers based on electrowetting [61], nonlinear electrokinetic effects [62] and acoustic streaming [63] are usually complicated to fabricate. However, simple, portable, hand-powered mixer that exploits movement of bubbles in microchannels was developed by Garstecki and co-workers [64]. More complicated procedures are needed to incorporate (especially on polymer-based devices) metal building components into microfluidic systems, for applications such as on-chip heating and magnetic sorting. A microsolidics method has been developed to fabricate complex metallic structures (like microheaters or magnets) by injecting liquid solder into microfluidic channels, and allowing the solder to cool and solidify [65, 66] (Fig. 2).

**Fig. 3:**
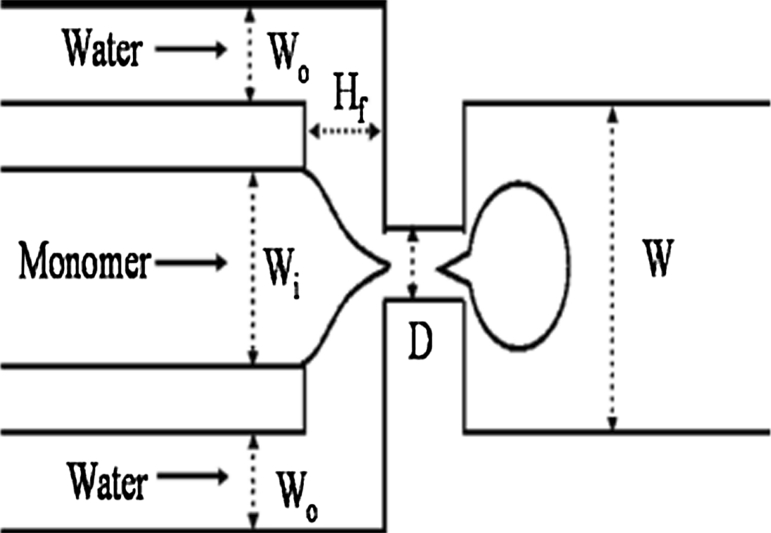
Typical scheme of flow-focusing microfluidic device. An orifice is placed at a distance Hf = 250 μm downstream of three coaxial inlet streams. Water is supplied to the two side channels which have widths Wo = 120 μm; monomer is forced into the central channel which has a width Wi = 100 μm. The width of the orifice is D = 80 μm; the width of the downstream channel is W = 240 μm (Nie et al. 2008 [68]; with kind permission from Springer Science + Business Media)

Microsolidics simplifies the incorporation of metals into microfluidic channels, but has several disadvantages. They can only be used with metals (or alloys) characterized by low-melting point (<300 °C) and affinity for the surface of the channel wall. These solders are usually more expensive than commonly used, and some are not biocompatible, particularly those that contain heavy metals like Pb or Cd. This method cannot be used to fill “dead-end” channel, and it is currently difficult to use this process to fabricate wires with cross-sectional dimensions <10 μm [65, 66].

**Fig. 4:**
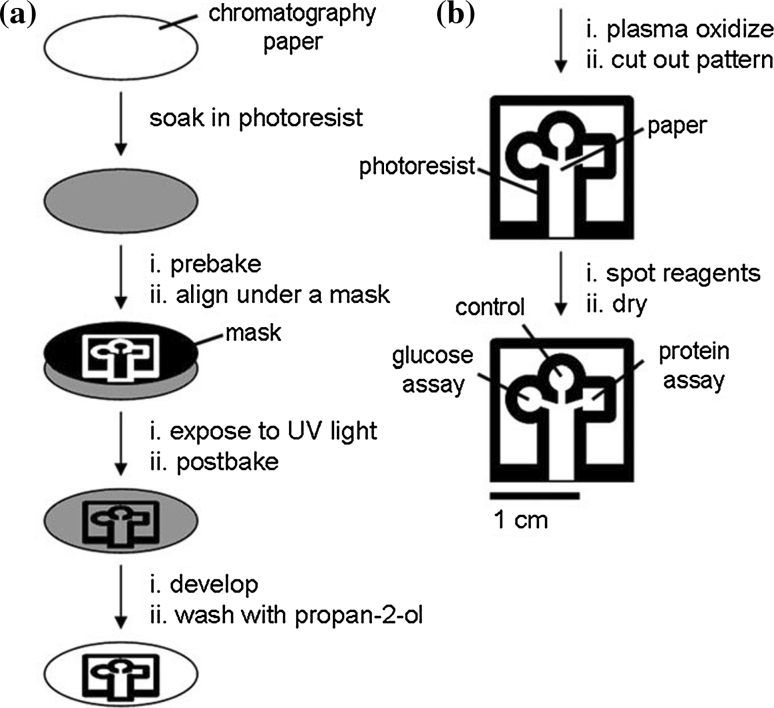
Fabrication of paper-based microfluidic device using photolithography technique (described by Martinez et al. 2007 [42])

The other components used in microfluidic devices are droplet and bubble generators. It has been shown that droplet and bubble-based microfluidics can perform Boolean logic functions [67]. The use of immiscible fluids for the formation of emulsions in microfluidic devices in controlled, individual segments (droplets) enabled rapid mixing of fluids and is a potent high-throughput platform for biomedical and chemical research and applications. Droplet-based systems have been used to directly synthesize particles with diameters from several micrometers to hundreds of micrometers and encapsulate many biological entities for biomedicine and biotechnology applications [68, 69]. There are several ways to generate droplets and bubbles in microfluidic systems but two common methods that depend on the geometry of the channel namely the flow-focusing (Fig. 3) and the T-junction are commonly applied [69].

## μPADs Manufacturing Process

**Fig. 5:**
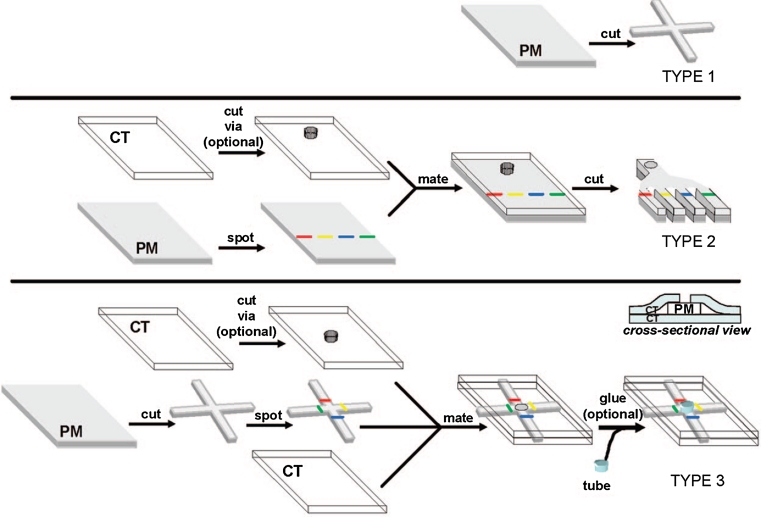
Paper cutting technique for fabrication of paper-based microfluidic device (reprinted with permission from Fenton et al. 2009 [75]; copyright (2012) American Chemical Society)

**Fig. 6:**
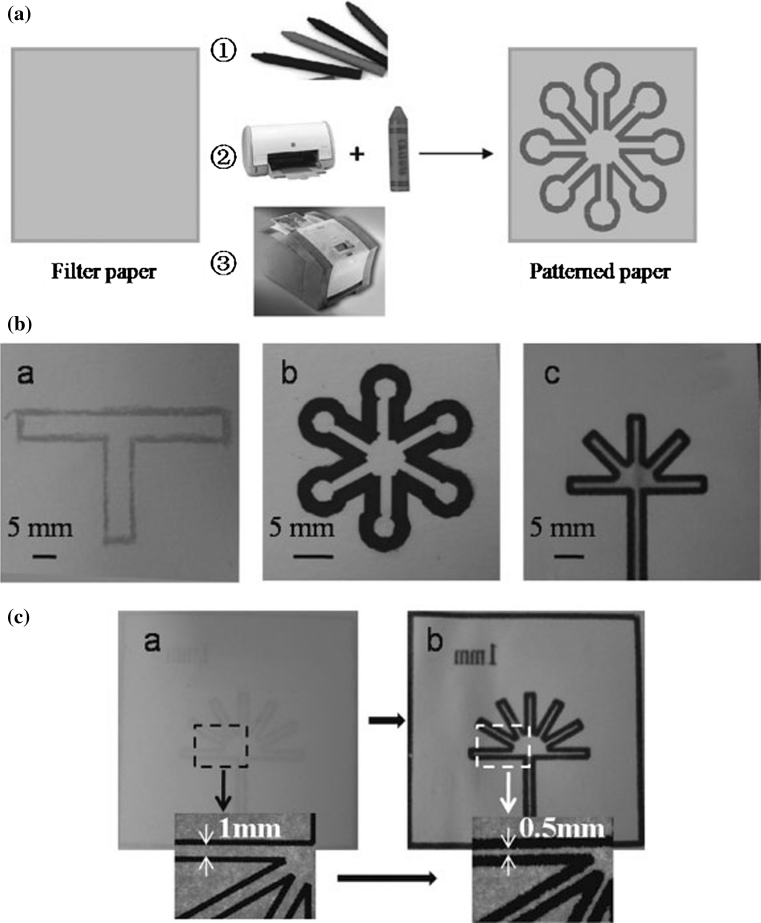
Schematic illustration of the processes to produce patterned paper with wax (described by Lu et al. [78])

On the other side, manufacturing process of the second group of microfluidic devices—Microfluidic paper-based analytical devices (μPADs) is very easy, cheap (estimated price is <$10 per square meter even for high-quality chromatography paper), and can be performed literally at home [2]. The first team that made such a device was a Whitesides Group of Harvard University [42] (Fig. 4). Recently, there are many techniques reported in the literature for fabricating paper-based microfluidic devices including photolithography [42, 70, 71], plotting with an analogue plotter [72], ink jet etching [73], plasma treatment [74], knife cutting [75, 76] (Fig. 5), wax printing [77, 78] (Fig. 6) and variation (wax screen printing) [79], ink jet printing [80] flexography printing [81] and laser treatment [82]. The main objective of those techniques’ applications is to create hydrophobic barriers on sheet of hydrophobic cellulose that constitute the walls of millimeter-sized, capillary channels. To prevent leakage and to keep the applied solution in the channels, paper strip may be surrounded by a polypropylene material, for example a stick tape or may be hidden in plastic cover [44]. To create hydrophilic microchannels on paper, a variety of hydrophobic substances are used such as photoresist SU-8, wax or as alkyl ketene dimer (AKD) ($0.1, $0.01 and $0.00001, respectively, for patterning filter paper of 100 cm^2^). Depending on the hydrophobic agents, the paper pores can be blocked (after using SU-8 or PDSM), covered by physical deposition (polystyrene or wax) or cellulose fibers can be modified chemically (after using AKD) [44]. After chemical modification, paper hydrophobicity cannot be removed by organic solvent extraction [83]; paper hydrophobicity caused by physical deposition can be largely removed by organic solvent washing, making it possible to use organic solvent etching methods to fabricate paper-based microfluidic devices [84].

## Flow Control in μPADs

**Fig. 7:**
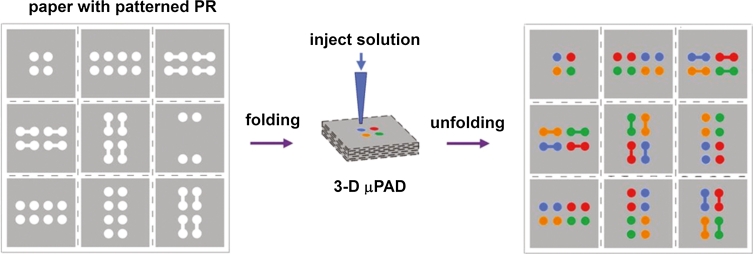
Three-dimensional paper microfluidic devices assembled using the principles of origami (reprinted with permission from Liu and Crooks, 2011 [87]; copyright (2012) American Chemical Society)

A number of valving mechanisms have been developed for controlling the pressure-driven flow of fluids in conventional μTAS; however, these technologies cannot be applied to μPADs in which the movement of fluids is based on capillary flow. To achieve multi-steps in analysis and diagnostic procedures (e.g. premixing or filtering samples, controlling fluid flow), improve sensitivity and separation selectivity changes made by the researchers refer to both the spatial structure and materials used for their production. For example, Li and co-workers (2008) [74] designed cellulose mechanical switches, filters and separators on μPADs made by plasma treatment. One of the solutions is hydrophobic paper strip with a small hydrophilic area matched with hydrophilic channel in device and is simply activated by mechanical pulling [74, 85]. Other options to achieve the above-mentioned purposes are μPADs designed in 3D technique, which enable the transport of fluids both vertically and laterally from a single inlet to numerous detection zones [86]. Three-dimensional μPADs offer several potential advantages over 2D devices. They can incorporate intricate networks of channels connected to large arrays of test zones, and each layer in a 3D μPAD can be made of a different paper. Therefore, multiple functionalities provided by different types of paper can be combined into a single device [2]. Interestingly, Liu and Crooks [87] developed μPAD using origami principles (Fig. 7). Whitesides group [88] reported a device with buttons for connecting and disconnecting the fluid flow between channels. Chen et al. [89] created a fluidic diode (two-terminal component that promotes or stops wicking along a paper channel) and functional circuit to manipulate two fluids in a sequential manner.

## Detection Techniques, Separation Methods and Applications of Microfluidic Devices

All primary detection methods and their variations have been successfully integrated or coupled with μTAS devices, including optical detectors based on UV–Vis light, chemiluminescence and fluorescence. Moreover, electrochemical detectors, magneto-resistive sensors (GMR), mass spectrometric (MS) and nuclear magnetic resonance (NMR) [13, 40] have been extensively applied (see Table 1). Four detection methods have been reported for the detection of analytes in paper-based microfluidics: colorimetric, electrochemical (EC) [90], chemiluminescence (CL), and electrochemiluminescence (ECL), but most studies have been focusing on colorimetric detection (which is typically related to enzymatic or chemical color-change reactions) and EC detection. CL and ECL performed in the dark and are independent of ambient light and also are the most common optical detection methods in microfluidics area [44, 91]. However, they have not been widely used in μPADs [82, 92].

In recent years, all the basic fluidic manipulations (mixing, diluting, etc.) have been adapted to microfluidic chips [93]. LOC connected with micellar electrokinetic chromatography (MEKC) and electrochromatography (CEC) have been applied to the separation of various components in multidisciplinary field, but techniques based on microchip capillary electrophoresis (MCE) are the most common methods integrated with μTAS devices and many studies were based on it. Practical applications of that separation protocols are widely used in many studies in genetics and molecular biology, including polymerase chain reaction (PCR) [94] amplification of DNA in nanoliter droplets [95, 96], reverse transcription (RT)-polymerase chain reaction (PCR) [97, 98], DNA extraction and real-time PCR for rapid pathogen identification [97, 99, 100], simultaneous DNA amplification and detection [101], removal of PCR inhibitors [102], mRNA isolation and amplification [103], mRNA quality and quantity control, nucleic acid detection, amplification and purification [104, 105], single cells genetic research and manipulations [106–109], gene expression research [108, 110], forensic DNA analysis [17] and many others. In molecular biology, the ability to manipulate and analyze single cells is important to understand the molecular mechanisms underlying cellular function [111].

In medicine field, they are used for neurotransmitters detection, cancer diagnosis and treatment [112, 113], cell and tissue culture growth and amplification [114–116], drug discovery and determination [19, 117, 118], detection and identification of microorganisms, pathogens [10, 11, 97, 99, 119] and proteins [12]. LOC devices combined with different detectors have been applied to detect environmental pollutants in order to separate metal ions [120–122], phenols [123, 124], explosives [125, 126] nitroaromatics [127], organic peroxides [128], and other environmentally relevant substances [15, 40, 93]. Most of these applications were performed in aqueous solution, except for one nonaqueous MCE example developed by the group of Collins [120] for measuring six toxic metal cations including Cd^2+^, Pb^2+^, Cu^2+^, Co^2+^, Ni^2+^, and Hg^2+^.

In recent years, the second group of microfluidic devices—μPADs are a subject of research activities, mostly for biochemical analysis but also for medical and forensic diagnostics. In the laboratory, paper filters are commonly used for chromatography and filtration purposes. One of the first paper-based diagnostic devices created was for urinalysis [42]. These devices utilize colorimetric assays to measure glucose and protein concentration in urine. Carrilho et al. [129] designed paper-based plates as a low-cost alternative to the conventional plastic microliter plates. This allowed mixing of different analytes for different assays that were not possible in a plastic plate. Another important application for paper-based devices is pathogen and toxin detection. One of the first functioning paper-based detection devices had been developed by Brennan’s research group—the paper-based device was able to detect neurotoxins paraoxon and aflatoxin B1 within five minutes at low concentrations, ~100 and ~30 nM, respectively [130]. Lateral flow paper chromatography and vertical flow diagnostic sensors based on bioactive paper are recently used to determine human blood type [8, 131–134]. Bioactive paper strips are also applied in genetic and biochemical analyses, like DNA detection [135] or ELISA tests [136, 137].

Currently, most paper-based devices utilize colorimetric assays, although there have been reports of electrochemical sensing in paper-based devices for detection of glucose, lactate, and uric acid in biological samples [90] and heavy metal detection in water [138, 139]. In this work, a novel method of electrokinetic sensing in a paper-based microfluidic device was proposed. Chemiluminescence [82] and electrochemiluminescence [92] detection techniques are described; however, they have not been widely used in μPADs. Several studies on bioactive paper [130, 135–137, 140] have proposed a few promising ideas to enhance the sensitivity and selectivity of the colorimetric detection for paper-based microfluidic devices.

## LOC Development and Future Trends

**Fig. 8:**
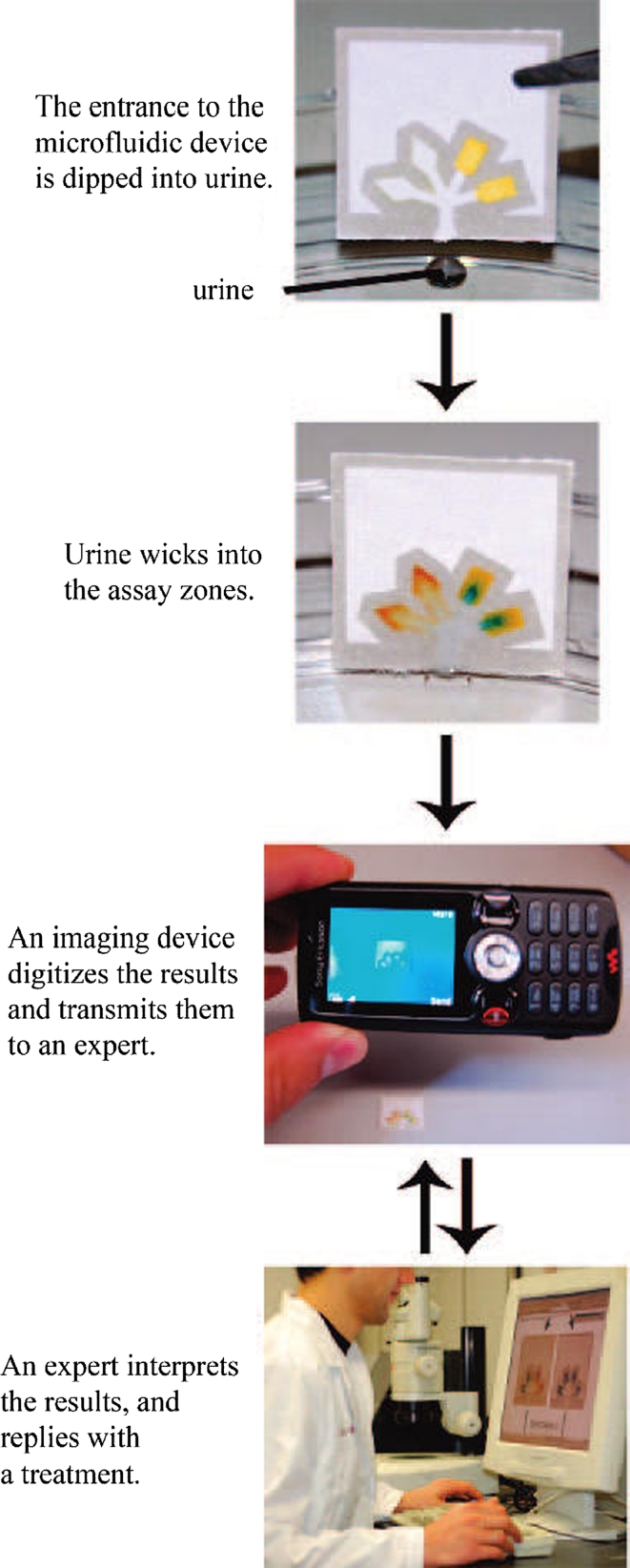
General strategy for performing inexpensive bioassays in remote locations and for exchanging the results of the tests with offsite technicians (reprinted with permission from Martinez et al. 2008 [70]; copyright (2012) American Chemical Society)

Most important for our future is environmental protection and ensuring the people health. Healthcare procedures (monitoring, control, prevention) and diagnostic tests are expensive. In poor and development countries infectious diseases, that would be treatable in a developed nation, are often deadly. Healthcare clinics, even if they have drugs to treat a certain illness, often suffer from the lack of diagnostic equipment and qualified staff. This creates a need to develop low-cost, simple to-use, point-of-care (POC) diagnostic methods for diagnosis and monitoring the treatment of patients [141]. The same problem—lack of diagnostic tools—exists in case of environmental monitoring. Lab-on-chip technology can be important and vital component of efforts to improve both a global health through the development of point-of-care testing devices [141] and environmental protection trough development analytical devices for environmental samples. In genetic research and molecular biology field, understanding the cell biology will become accessible through high-throughput single-cell data analysis. For example, multiplex quantitative polymerase chain reaction (qPCR) is limited in the number of reactions. If we wish to measure 100 genes from 100 cells, we need 10,000 reactions. Microfluidic chips can be used to overcome these limitations by combinatorially mixing the samples and gene detectors and by performing thousands of reactions in parallel on a single chip and can provide high mRNA-to-cDNA efficiency and decreased risk of contamination [110, 142]. It is also possible to isolate and amplify single chromosomes from a single cell. Fan et al. [109] developed a microfluidic device capable of separating and amplifying homologous copies of each chromosome from a single human metaphase cell in independent chambers. This enabled them to study the two alleles (or haplotypes) of each chromosome independently. This method can be used to obtain accurate haplotype information in personal genome sequences, to understand meiotic recombination and to directly study the human leukocyte antigen haplotypes of an individual. Molecular biologist needs particular devices for rapid isolation and characterization of single cells from small biological specimens. Future challenges include sensitive, high-throughput and simple-to-use microdevices for characterizing proteins, signaling, epigenetic, and metabolic states in single cells, and correlating these measurements with physiological characteristics. Existing techniques for whole-genome/trancriptome amplification prior to sequencing suffer from bias and non-specific products that have to be characterized and eliminated. In particular, there is a need to develop methods for multiplexing samples and precise unbiased counting of molecules, which is possible with using microfluidic devices. Depending on the chip platform being used, several thousand to several hundred thousand distinct oligonucleotides can be synthesized on a single chip. In principle, these massive parallel microarrays can reduce the cost of oligonucleotides by orders of magnitude [105]. In developed countries, there are many valued features of diagnostic tools, including speed, sensitivity and specificity. In developing countries, with limited resources, where the healthcare infrastructure is less well developed, many difficulties must be overcome to apply the LOC device; therefore, ease of use must also be considered. In this case, paper-based devices can be extremely useful. New techniques for making paper devices and their use in clinical and environmental laboratory will be evaluated in accordance with the principles of economics—mainly in terms of material costs and production in mass production—the utility and ease of use. Their advantage is the simplicity of design and ease of interpretation of test results. In contrast to more complicated LOC devices, they are self-contained and independent from the other devices. These features also do not exclude the possibility of cooperation μPADs with other units. Their compatibility to telemedicine, particularly with mobile phone transmission or interpretation of test results is study [70, 143]. Whitesides’ group in Harvard University showed that an image of the device can be taken by a camera on a mobile phone and then sent to a remote location for analysis [70] (Fig. 8). Liu and Crooks [144] reported, for point-of-care diagnosis, microelectrochemical biosensing platform that is based on paper fluidics and powered by an integral metal/air battery. Vella and co-workers [145] described micropatterned paper device designed for blood from a fingerstick uses to measuring markers of liver function. These studies are just beginning to show the potential for paper-based diagnostic devices for developing countries. However, paper-based microfluidic devices that rely on complicated instrumentation for result interpretation may only have value for laboratory uses [44]. The possibility of using paper in preconcentration is obvious, since it is already widely used in chromatographic applications and is much cheaper than conventional materials used in SPE. Furthermore, paper spray ionization has been recently reported as a direct sampling ionization method for mass spectrometric analysis [146]. Thus, the use of paper as a platform for preconcentration and mass spectrometric analysis can be an excellent low-cost alternative to the conventional analytical methods for trace compounds. The combination of paper spray with miniature mass spectrometers offers a powerful impetus to wide application of mass spectrometry in non-laboratory environments [146]. μPADs area research is still at an early stage and significant efforts will be needed to nurture it into a more matured platform technology in diagnostic, point-of-care (POC), and environmental monitoring applications [44].
